# Regulation of the sensitivity of hepatocarcinoma cells by ORMDL3, to sorafenib by autophagy

**DOI:** 10.1007/s12032-022-01767-z

**Published:** 2022-08-16

**Authors:** Yixiao Sun, Xueran Guan, Ting Zhang, Yue Li, Huiling Shi, Ashleigh Tinotenda Chitakunye, Hanyu Hong, Shihui Zhang, Qin Zhu, Lin Cai

**Affiliations:** Department of Biopharmaceuticals, School of Pharmaceutics Sciences, Wenzhou Medical University, Chashan, Wenzhou, 325035 Zhejiang Province China

**Keywords:** ORMDL3, Sorafenib, Autophagy, Hepatocellular carcinoma, Oxidative stress

## Abstract

Serum orosomucoid1-like protein 3 (ORMDL3) is a membrane protein in the endoplasmic reticulum, known to regulate many important signal transduction processes and autophagy regulation, but it is unclear whether it is involved in the intratumoral microenvironment and cancer drug resistance. Our present study found that silencing ORMDL3 increases the inhibitory effect of sorafenib on the viability and proliferation in HCC cells, and increases the sensitivity of HCC cells to sorafenib. In addition, silencing ORMDL3 can increase ROS levels by inhibiting autophagy, thereby increasing sorafenib-induced apoptosis of HCC cells. Further, our study also found that ORMDL3 silencing inhibits autophagy through the PERK-ATF4-Beclin1 pathway, thus affecting sorafenib sensitivity. The in vivo effects of sorafenib were tested by xenografting using nude mice. It showed that silencing ORMDL3 in HCC cells could increase the inhibitory effect of sorafenib on the growth of tumors. This is the first report to describe the relationships among ORMDL3, autophagy, and sorafenib resistance. This study provides available targets that might have a synergetic effect with sorafenib.

**Supplementary Information:**

The online version contains supplementary material available at 10.1007/s12032-022-01767-z.

## Introduction

Hepatocellular carcinoma (HCC) accounts for 75 to 85% of primary liver cancer and is the sixth most common malignancy worldwide and the fourth leading cause of cancer death worldwide [1]. Since this disease is usually diagnosed at an advanced stage, its prognosis remains unsatisfactory. Currently, the multi-kinase inhibitor, sorafenib, is one of the few drugs approved for the treatment of advanced HCC [2, 3]. However, experimental studies have shown that the median survival time of HCC patients treated with sorafenib was only 2 to 3 months longer than that of the placebo group because HCC cells develop resistance to sorafenib under the influence of various factors, thus affecting the efficacy [4, 5]. Studies have shown that genetic heterogeneity of HCC, tumor microenvironment, changes in PI3K/AKT pathway, epithelial–mesenchymal transition (EMT), autophagy activation, and other factors can affect the resistance of HCC cells to sorafenib [6, 7]. Therefore, studying the underlying mechanism of sorafenib resistance and providing a new method for combination therapy of advanced HCC is still a great challenge.

In oncology, autophagy is considered to be a double-edged sword: on one hand, it is an environment-dependent tumor inhibition mechanism, on the other hand, it can also promote the survival of tumor cells under stress and drug resistance conditions [8–10]. There are many studies on the inhibition of autophagy enhancing the antitumor effect of the multi-kinase inhibitor, sorafenib, in HCC, and further studies have shown that autophagy inhibition enhances the lethality of sorafenib, mainly through endoplasmic reticulum stress (ERS)-related apoptosis [11–13]. Moreover, the reactive oxygen species (ROS) level of HCC cells treated with sorafenib reaches a high level, resulting in apoptosis and necrosis of HCC cells. However, the higher antioxidant capacity of HCC cells than normal cells can reduce ROS-induced cell death [14]. Taken together, these studies suggest that targeting autophagy is an effective strategy for the treatment of liver cancer and provides the basis for future clinical trials to explore this combination therapy in the treatment of tumors.

ORMDL3 gene is located in the q2 region of chromosome 17 and is a susceptibility gene for asthma [15, 16]. ORMDL3 protein is an endoplasmic reticulum (ER) membrane protein, which plays an important role in oxidative stress, inflammation, ERS, unfolded protein response (UPR), and sphingolipid metabolism [17, 18]. ORMDL3 can inhibit the rate-limiting enzyme of sphingolipid biosynthetic, serine palmitoyltransferase (SPT), thus, regulating the metabolism of sphingolipids from the source [19]. Cancer cells can further support their proliferation, metastasis, and resistance to chemotherapeutics by down-regulating pro-cell death sphingolipids like ceramide [20]. Previous studies showed that ORMDL3 knockdown markedly increased the ceramide precursors in HepG2 hepatocarcinoma cells. Moreover, in the irritant-induced sterile inflammation mice model, ORMDL protein expression was obviously down regulated with increasing ceramide levels in vivo [21]. However, the relationship between ORMDL3 and cancer needs more exploration. Studies have shown that the ORMDL3 gene is also involved in the regulation of autophagy. For example, ORMDL3 can act as a negative regulator of antigen-mediated mast cell activation through the ATF6-UPR autophagy-dependent pathway [22]. HCC cells may resist various stimuli caused by sorafenib treatment through autophagy, maintain cell homeostasis, and avoid death. In this study, the regulation of ORMDL3 gene expression that might affect the sensitivity of HCC cells to sorafenib treatment was first examined, and the particular mechanism of inhibiting autophagy through the PERK-ATF4-Beclin1 pathway was elucidated.

## Material and methods

### Reagents and antibodies

Primary antibodies applied in this study include antibodies against ORMDL3 (Abcam, UK, ab211522), Bax, Bcl-2, PARP, Caspase-3, Caspase-9, P62, Beclin1 (Proteintech-Group, Wuhan, China, 50599-2, 12789-1, 1337-1, 19677-1, 10380-1, 66184-1, 11306-1), LC3B (Novus-Biologicals, USA, NB100-2220), ATF4 (Cell Signaling Technology, D4B8, Danvers, USA), PERK, Phospho-PERK (Bioworld Technology, Shanghai, China, BS2156, BS66100), Goat anti-Rabbit IgG-HRP H&L, Goat anti-Mouse IgG-HRP H&L (Bioworld Technology, Shanghai, China, BS13279, BS12478), Alexa Fluor®488 Goat Anti-Rabbit IgG H&L, Alexa Fluor®488 Goat Anti Mouse IgG H&L(Abcam, UK, ab150077, ab150113). 3-(4,5-dimethylthiazol-2-yl)-2,5-diphenyltetrazolium bromide (MTT), BCA Protein Assay Kit, BeyoECL Plus, DAPI Staining Solution, Apoptosis and necrosis Assay Kit, Annexin V-FITC/PI Apoptosis Detection Kit, Reactive Oxygen Species Assay Kit, and ATP detection Kit were purchased from Biyuntian Institute of Biotechnology (Nanjing, China). MitoSOX Red Mitochondrial Superoxide Indicator was purchased from ThermoFisher Scientific (Shanghai, China).

### Cell culture and transfection

HepG2, SMMC-7721 hepatoma cell lines, and normal human HL-7702 liver cell lines were purchased from the Shanghai Cell Bank (Chinese Academy of Sciences, Shanghai, China). The SMMC-7721 cell line was used in most of the experiments, and short tandem repeat (STR) analysis was performed on the cell line to determine its origin from HCC. Cells were grown in Roswell Park Memorial Institute-1640 medium containing 10% fetal bovine serum (FBS, Gibco, MD, USA), 100 U/mL penicillin G, and 100 μg/mL streptomycin, and incubated at 37 °C in a humidified incubator containing 5% CO_2_.

### siRNA and plasmid transfections

The lentiviruses with scrambled shRNA against ORMDL3 (Lv-shORMDL3) and the shRNA control (Lv-shCon) were constructed in GV248 (Genechem, Shanghai, China). The ORMDL3 overexpressed plasmid was constructed in GV362, with pcDNA3.1-CMV-GFP plasmid as the backbone (Genechem, Shanghai, China) [23]. SMMC-7721 cells were plated and grown in 6-well plates before transfection at approximately 60% confluence. According to the manufacturer's protocol, the cells were transfected with siRNA targeting ORMDL3, and the overexpressed cells were transfected with a plasmid encapsulated with Lipofectamine® 2000. After culturing for 48 h, the transfected cells were screened with puromycin (4 μg/mL) for 2 weeks to form a stable transduction reagent pool. The transfected cells were used for subsequent experiments.

### Cell viability assay

SMMC-7721 cells were treated with trypsin, counted, and then plated in a 96-well plate at 10,000 cells/well. After 24 h of treatment with sorafenib at 4 μM, 20 μL of MTT was added to each sample, and the plate was placed in the incubator for another 4 h. Next, the supernatant was discarded, 120 μL of DMSO was added to each well, and the plate was shaken for 10 min in the dark to sufficiently dissolve the formazan crystals. Then, the absorbance at 490 nm was measured with SpectraMax 190 (Molecular Devices, USA). Three independent experiments were performed and the data were statistically analyzed.

### Total RNA isolation and quantitative real-time PCR

Total RNA was isolated using Trizol RNA extraction reagent (Life Technologies, Grand Island, NY) and reverse transcribed into cDNA using Superscript III first chain synthesis system (Invitrogen, China). SYBR Green PCR Master Mix (Vazyme, China) was used to prepare the PCR reaction mixture. The primers were used for qRT-PCR as follows:

Forward, 5'-AACACGCGGGTGA TGAACAG-3',

Reverse, 5'-AGGGACACTCACAAACGGGA-3' for human ORMDL3 gene;

Forward, 5'-GGAGCGAGA TCCCTCCAAAA T-3',

Reverse, 5'-GGCTGTTGTCA TACTTCTCA TGG-3' for human GAPDH gene.

The QuantStudio-Q3 Detection System (ABI, Singapore) performed a thermal cycle at 42 °C for 2 min, then at 95 °C for 15 s, 60 °C for 60 s, 95 °C for 1 s with 40 cycles of amplification. Relative expression was evaluated using the competitive critical threshold (ΔΔCt) method using GAPDH as the reference gene. Three independent experiments were performed.

### Western blot

SMMC-7721 hepatoma cells were lysed on ice for 15 min and then collected into a centrifuge tube and centrifuged (12,000 rpm, 4 °C) for 15 min. The protein content of the supernatant was determined by BCA. Purified protein samples were incubated with 4× loading buffer and heated at 95 °C for 10 min. Protein samples from each group were isolated by SDS-PAGE and transferred to PVDF membranes under constant electric current (Amersham hybond-P, GE Healthcare, Buckinghamshire, UK). The membrane was sealed with 5% skim milk for 90 min and then incubated overnight with the primary antibody at 4 °C, followed by incubation with secondary antibodies for 1 h and visualized with an ECL Plus Western Blotting Detection System (GE Healthcare). Films were scanned or photographed and molecular weights and net light density of target bands were analyzed using a gel image processing system (Image lab). Each experiment was repeated independently more than three times, and then quantitative analysis was performed.

### Colony formation assay

SMMC-7721 cells were treated with trypsin, counted, and then plated in a 6-well plate at 1000 cells/well. After 24 h of treatment with sorafenib at 4 μM, the SMMC-7721 cells were cultured for 12 days and fixed with 4% paraformaldehyde for 30 min. After drying, 1 mL of crystal violet staining solution (Biyuntian Institute of Biotechnology, Nanjing, China) was added, shaken evenly, dyed for 5 min, air dried naturally, and then photos were taken. The colonies were counted and the clone formation rate of each group was compared. The experiment was repeated three times for statistical analysis.

### Apoptosis assay

SMMC-7721 hepatoma cells were seeded into a 6-well plate with 200,000 cells per well. Apoptosis levels were detected using the PI/Hoechst double-staining kit. 1 mL of the prepared staining solution (PI staining solution:Hoechst staining solution:cell staining buffer = 5 μL:5 μL:1 mL) was added to each well, shaken well, wrapped with aluminum foil to avoid light, and incubated for 30 min at 4 °C. Then, the fluorescence was observed with an inverted fluorescence microscope (Nikon, Japan) and the image was collected. Each experiment was independently repeated three times.

### Annexin V-FITC/propidium iodide (PI) apoptosis assay

Annexin V-FITC and PI were detected by flow cytometry using two channels to determine the proportion of apoptotic cells. The drug-treated suspension and adherent cells were collected into a flow tube and incubated with 5 μL Annexin V-FITC and 10 μL PI in a dark room at room temperature for 15 min. Data were obtained using Beckman Cytoflex LX flow cytometry (Beckman Coulter, USA) and processed using CytExpert software (Beckman Coulter, USA). Each experiment was repeated three times independently, and the data were statistically analyzed.

### Measurement of intracellular reactive oxygen species

SMMC-7721 cells were seeded into a 6-well plate with 200,000 cells per well. The fluorescent probe DCFH-DA (dichlorodihydrofluorescein acetoacetate) was used to detect reactive oxygen species. The DCFH-DA was diluted in serum-free medium at 1:1000 and incubated at a final concentration of 10 mM/L, 37 °C, and 5% CO_2_ for 20 min. After treatment, data were obtained using Beckman Cytoflex LX flow cytometry (Beckman Coulter, USA) and processed using CytExpert software (Beckman Coulter, USA). Each experiment was performed in triplicate.

### Immunofluorescence

SMMC-7721 cells were uniformly seeded on sterile cover slips in 6-well culture plates for adherent growth. After the indicated treatment for a specified time, the cells were sequentially fixed in 4% paraformaldehyde and permeabilized with 0.5% Triton X-100, and then, the coverslips were immersed in 5% BSA at room temperature for 1 h. Subsequently, the pretreated samples were incubated with primary antibodies (dilution 1:100) overnight at 4 °C and then with goat anti-mouse/rabbit secondary antibody (dilution 1:100) in the dark for 2 h. Finally, following incubation with DAPI for nuclear staining. Confocal microscopy (Nikon, Japan) was used to observe cell fluorescence and image collection was carried out. Image J software was used to analyze cell fluorescence intensity. Five regions were randomly selected from each sample for evaluation and each experiment was repeated independently for three times for statistical analysis.

### Immunohistochemical analysis

The tissue sections were dewaxed, hydrated, and heated in 0.01 mol/L sodium citrate buffer for antigen extraction, and then incubated with 3% hydrogen peroxide to block endogenous peroxidase activity. The tissue microarray was then incubated with the specific primary antibody for 1.5 h at room temperature or 4 °C overnight, followed by the corresponding secondary antibody and streptomycin anti-biotin protein. Subsequently, DAB was used for color development, DAPI and hematoxylin were used for dyeing, alcohol was used for hydrating, and xylene was used for transparency. The plates were sealed with neutral resin. The stained tissue sections were observed with a forward microscope (Nikon, Japan), and the images were collected. Image Pro Plus 6 software was used to calculate the mean optical density (AOD) of the five regions of each sample for quantitative analysis.

### In vivo experiment

Animal researches were carried out in compliance with the National Institutes of Health's Laboratory Animal Care and Use Guidelines, and approved by the Institutional Animal Care and Use Committee of Wenzhou Medical University (license no. wydw 2021–0619).

Male BALB/C-nu nude mice (6 weeks) (WeitongLihua Laboratory, Zhejiang, China) were used in the experiment. The mice were divided into the shControl group, shORMDL3 group, shControl + Sora group, and shORMDL3 + Sora group. At least, 5 nude mice were randomly allocated to each group. Prepared SMMC-7721 cells of shControl or shORMDL3 (1 × 10^6^) were injected into the right axilla fossa of each mouse, respectively. Sorafenib (30 mg/kg) treatment or saline as control was initiated when tumor volumes reached 60–100 mm^3^ and delivered intraperitoneally every 2 days for 28 days [24]. Tumor size and body weight were measured every 2 days, and tumor volume was calculated according to the equation: volume (cm^3^) = L × W^2^/2 with L and W representing the largest and smallest diameters, respectively. On the 28th day, mice were euthanized by cervical dislocation, and then tumors were isolated from each mouse and prepared for subsequent experiments.

### Statistical analysis

The statistical analyses were performed using GraphPad Prism 7 software. All results are expressed as the mean ± SD of at least three independent trials. Differences between the two groups were analyzed using Student’s t test. One-way ANOVA was used for multiple groups to assess significant differences between study groups. *P* < 0.05 was considered statistically significant.

## Results

### Increase in the sensitivity of liver cancer cells to sorafenib by silencing ORMDL3

**Fig. 1:**
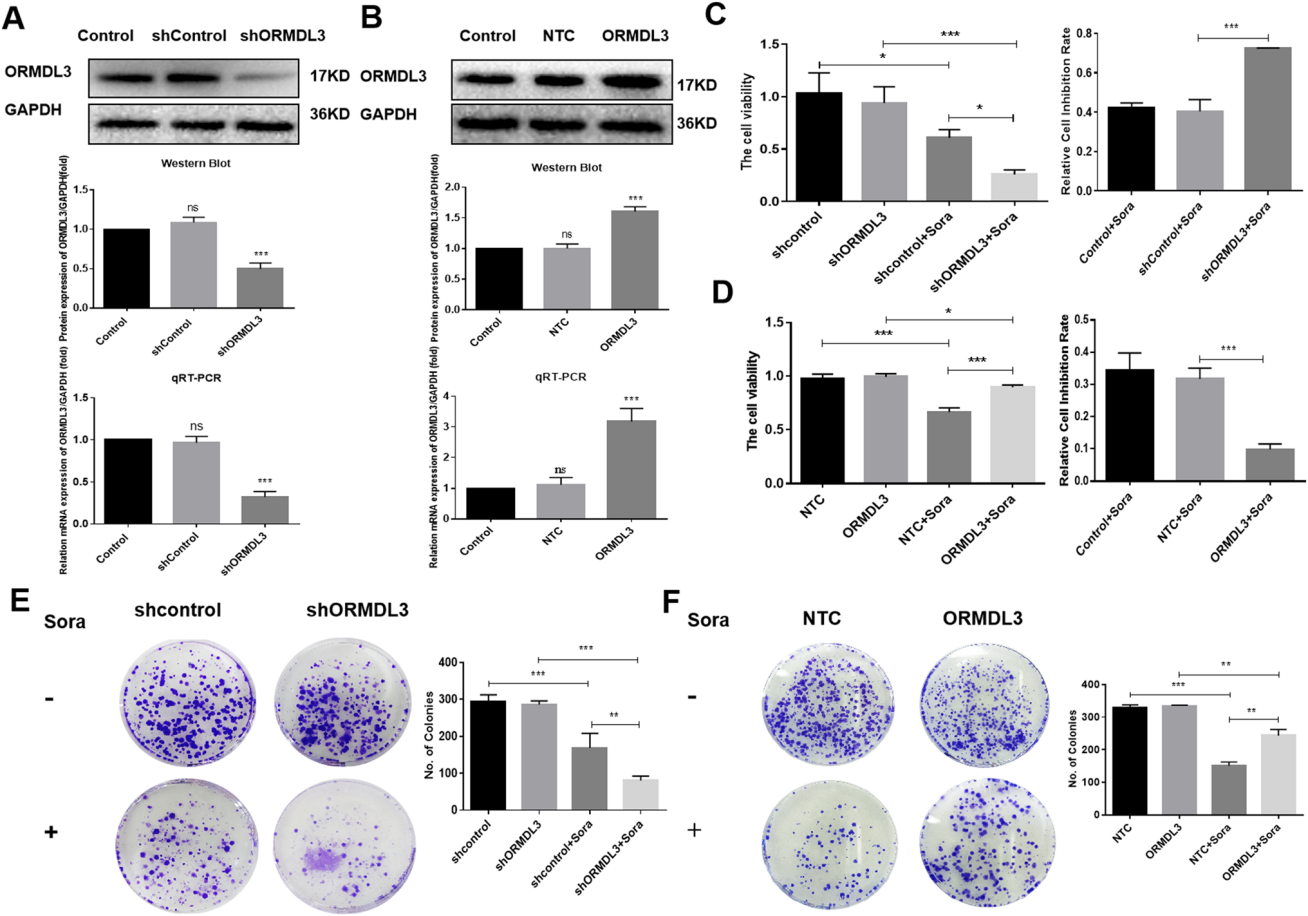
Increase in the sensitivity of liver cancer cells to sorafenib by silencing ORMDL3. **A**, **B** Western Blot and RT-qPCR were used to detect the expression of ORMDL3 at protein and mRNA levels in SMMC-7721 cells after ORMDL3 deletion or overexpression of ORMDL3. **C**, **D** MTT assay was used to detect the cell viability and inhibition rate of different groups of human hepatoma cell line SMMC-7721 with ORMDL3 silenced and ORMDL3 overexpression. **E**, **F** Clonal formation survival and statistical quantification of ORMDL3 silenced or overexpressing of ORMDL3 HCC cells with or without sorafenib. Statistical analysis was performed using one-way ANOVA. ^ns^*P* > 0.05, *P < 0.05, **P < 0.01, ***P < 0.001. The data are presented as the means ± SD (*n* = 3)

First, Western Blot and RT-qPCR were used to detect the expression differences of ORMDL3 in human normal liver cells HL-7702 and human hepatoma cells HepG2 and SMMC-7721 in the presence or absence of sorafenib (Fig. S1 A, B). The results showed that there was no significant difference in ORMDL3 protein and mRNA expression levels. We detected the expression of ORMDL3 at protein and mRNA levels after transfection (Fig. 1A, B). After being treated with sorafenib (4 μM) for 24 h, the viability of HCC cells was detected by MTT assay, and the cell inhibition rate was calculated. The results showed that cell viability was significantly inhibited after sorafenib treatment, and the liver cancer cell viability was lower in ORMDL3-silenced group, which is consistent with the higher cell inhibition rate in ORMDL3-silenced group (Fig. 1C). However, the cell viability of the overexpressed ORMDL3 group was higher than that of the overexpressed control group, and the cell inhibition rate was lower than that of the overexpressed control group (Fig. 1D). The plate cloning-formation assay suggested that silencing ORMDL3 with sorafenib (4 μM) treatment significantly suppressed cell proliferation, but enhanced in overexpressed ORMDL3 group when compared with the control group (Fig. 1E, F). Taken together, these data showed that silencing ORMDL3 can increase the inhibitory effect of sorafenib on the viability and proliferation of liver cancer cells, opening up new ideas for improving the sensitivity of sorafenib.

### Increase in sorafenib-induced apoptosis of HCC cells by silencing ORMDL3

**Fig. 2:**
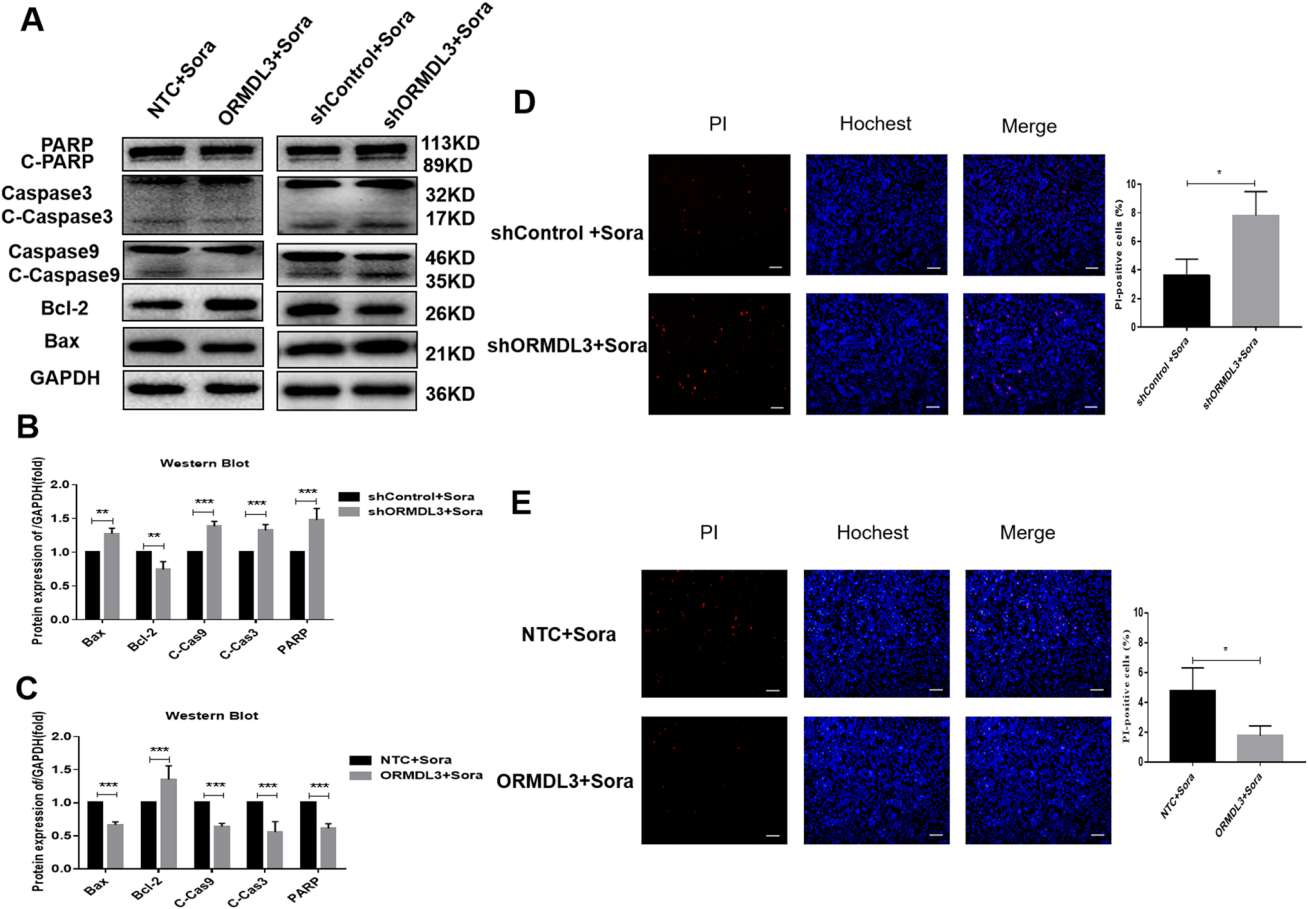
Increase in sorafenib-induced apoptosis of HCC cells by silencing ORMDL3. **A** Western Blot was used to analyze the expression levels of Caspase 3, Caspase 9, PARP, Bax, and Bcl-2 in SMMC-7721 cells after ORMDL3 was silenced or overexpressed under the action of sorafenib. **B**, **C** Quantitative analysis of Western Blot gray value. Statistical analysis was performed using one-way ANOVA. **P < 0.01, ***P < 0.001. The data are presented as the means ± SD (*n* = 3). **D**, **E** Silenced and overexpressed ORMDL3 hepatoma cells SMMC-7721 were treated with sorafenib (4 μM) for 24 h, and apoptosis and necrosis were detected. Representative images under ×100 inverted microscope after PI/Hoechst kit staining. Scale bar, 100 μM. Differences between the two groups were analyzed using Student’s *t* test. *P < 0.05. The data are presented as the means ± SD (*n* = 3)

Western Blot experiments were performed to detect the expression of hepatoma cell apoptosis-related proteins: Caspase9, Caspase3, PARP, Bax, and anti-apoptotic protein, Bcl-2. By using sorafenib (4 μM) treatment, as compared to the control group, Cleaved Caspase 9, Cleaved Caspase 3, and Cleaved PARP expressions were increased in ORMDL3-silenced HCC cells. The expression of apoptotic protein Bax increased and the expression of anti-apoptotic protein Bcl-2 decreased, indicating that the apoptosis of HCC cells was enhanced (Fig. 2A, B). On the contrary, the apoptosis of HCC cells overexpressed with ORMDL3 was weakened (Fig. 2A, C). PI/Hoechst double-staining kit was used to detect the effect of ORMDL3 on the apoptosis and necrosis of HCC cells, and it showed that after sorafenib (4 μM) treatment, ORMDL3 silencing increased the apoptosis and necrosis of HCC cells, while the overexpressed ORMDL3 showed the opposite effect (Fig. 2D, E). These data suggest that silencing ORMDL3 may increase the apoptosis of liver cancer cells induced by sorafenib.

### Increase in the apoptosis of sorafenib-induced HCC cells by silencing ORMDL3, by inhibiting autophagy

**Fig. 3:**
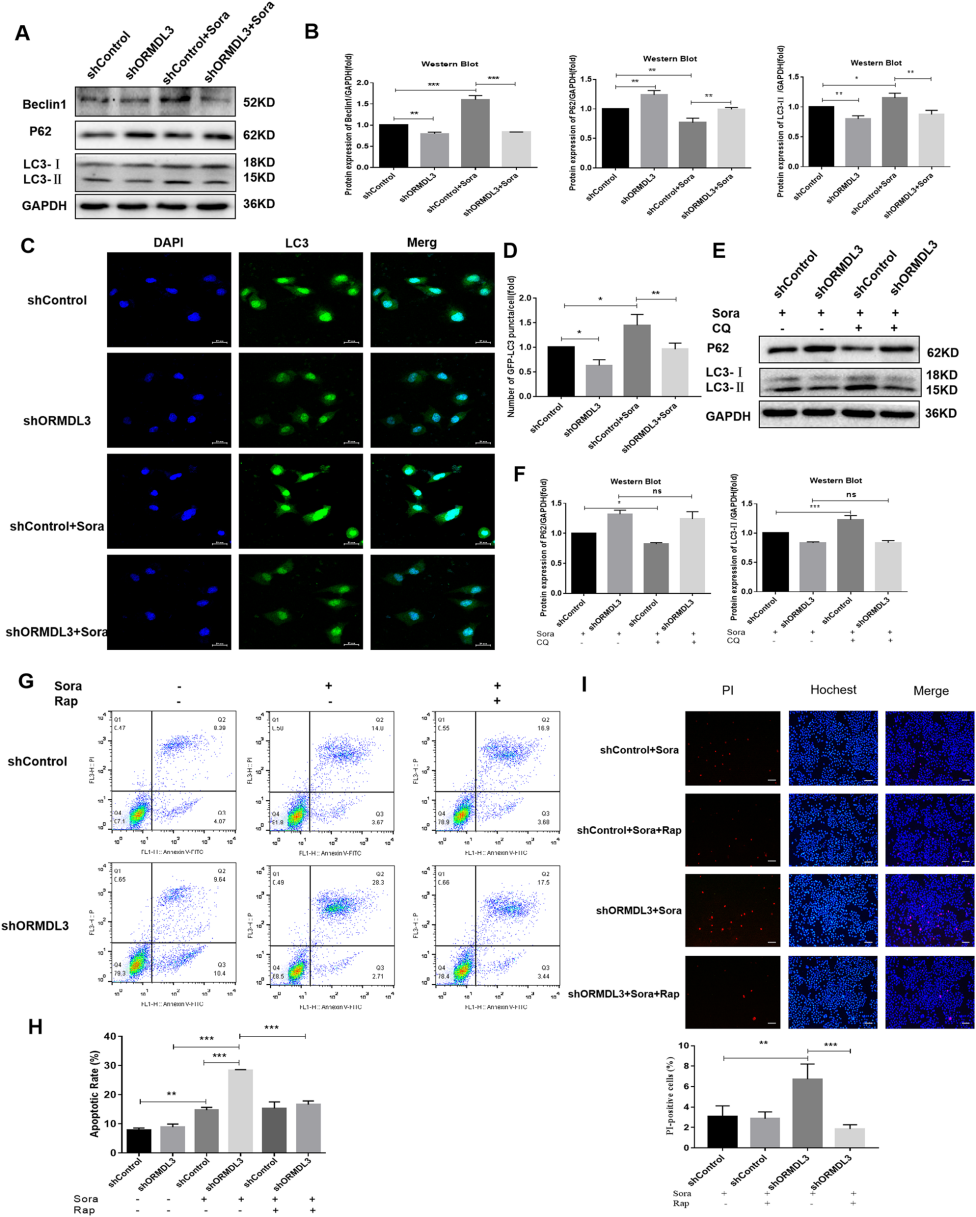
Increase in the apoptosis of sorafenib-induced HCC cells by silencing ORMDL3, by inhibiting autophagy. **A**, **B** Western Blot assay was performed to detect the expression levels of autophagy-related proteins LC3, P62, and Beclin1 in human HCC cells SMMC-7721 silenced by ORMDL3, and gray value analysis. **C**, **D** The expression of LC3 in HCC cells was detected by immunofluorescence. Scale bar, 25 μM. Five regions were randomly selected from each sample for evaluation. Statistical analysis was performed using one-way ANOVA. *P < 0.05, **P < 0.01. The data are presented as the means ± SD (*n* = 3). **E**, **F** Western Blot assay was used to detect the expression levels of autophagy-related proteins LC3, P62, and Beclin1 in ORMDL3-silenced human HCC cells SMMC-7721 combined with autophagy inhibitor CQ (50 μM), and gray value analysis. **G** Annexin V-FITC/PI Apoptosis Kit was used to analyze apoptosis levels in the ORMDL3-silenced group by flow cytometry. **H** Histogram of streaming data analysis. Statistical analysis was performed using one-way ANOVA. **P < 0.01, ***P < 0.001. The data are presented as the means ± SD (*n* = 3). **I** The apoptosis and necrosis of ORMDL3-silenced HCC cells SMMC-7721 were detected in the presence of sorafenib (4 μM) combined with Rap (100 nM), an autophagy agonist. Representative images under ×100 inverted microscope after PI/Hoechst kit staining. Scale bar, 100 μM. Differences between the two groups were analyzed using Student’s t test. **P < 0.01, ***P < 0.001. The data are presented as the means ± SD (*n* = 3)

To show whether the ORMDL3 gene can affect the autophagy of liver cancer cells, Western Blot assay was used to detect the expression of autophagy-related proteins: LC3, P62, and Beclin1. The results showed that the expression of autophagy markers LC3 and Beclin1 in ORMDL3-silenced liver cancer cells was significantly decreased, while the expression of substrate recognition factor P62 was increased. With the treatment of 4 μM sorafenib, silencing ORMDL3 gene can further inhibit the autophagy induced by sorafenib in HCC cells (Fig. 3A, B). Consistent with the above results, using immunofluorescence to detect changes in LC3 levels, the results showed that silencing ORMDL3 can inhibit the autophagy level of liver cancer cells, and can also inhibit the autophagy induced by sorafenib in liver cancer cells (Fig. 3C, D). Chloroquine diphosphate salt (CQ) is an autophagy inhibitor that inhibits autophagy degradation, resulting in a large accumulation of LC3 protein [25]. In the control group, LC3 protein accumulated and P62 expression decreased after CQ treatment. However, LC3 expression was not significantly increased in ORMDL3-silenced group under the combined action of sorafenib and CQ (Fig. 3E, F). The results showed that silencing ORMDL3 inhibits autophagy formation from the very beginning, thus, the inhibition of autophagy degradation did not cause a great accumulation of LC3.

It was further explored whether silencing ORMDL3 increases the apoptosis of HCC cells induced by sorafenib through inhibition of autophagy. The Annexin V-FITC/PI Apoptosis Kit was used to detect the apoptosis level of HCC cells by flow cytometry. The results showed that the apoptosis was significantly increased after sorafenib treatment, and the degree of apoptosis was more severe in ORMDL3 silencing group, whereas treatment with the autophagy activator, rapamycin (Rap), led to reduced apoptosis in ORMDL3 silencing group (Fig. 3G, H). The results of PI/Hoechst double-staining were consistent with flow cytometry. The apoptosis induced by sorafenib (red fluorescence) was enhanced in ORMDL3 silencing group; however, it was reversed by adding the autophagy activator, Rap (Fig. 3I). These results suggest that silencing ORMDL3 can inhibit the formation of autophagy, thus inhibiting the autophagy of sorafenib-induced HCC cells. Moreover, further findings show that silencing ORMDL3 can increase the apoptosis of sorafenib-induced HCC cells by inhibiting autophagy.

### Inhibition of autophagy of liver cancer cells by silencing ORMDL3, resulting in increased ROS levels, thereby promoting ROS-mediated apoptosis

**Fig. 4:**
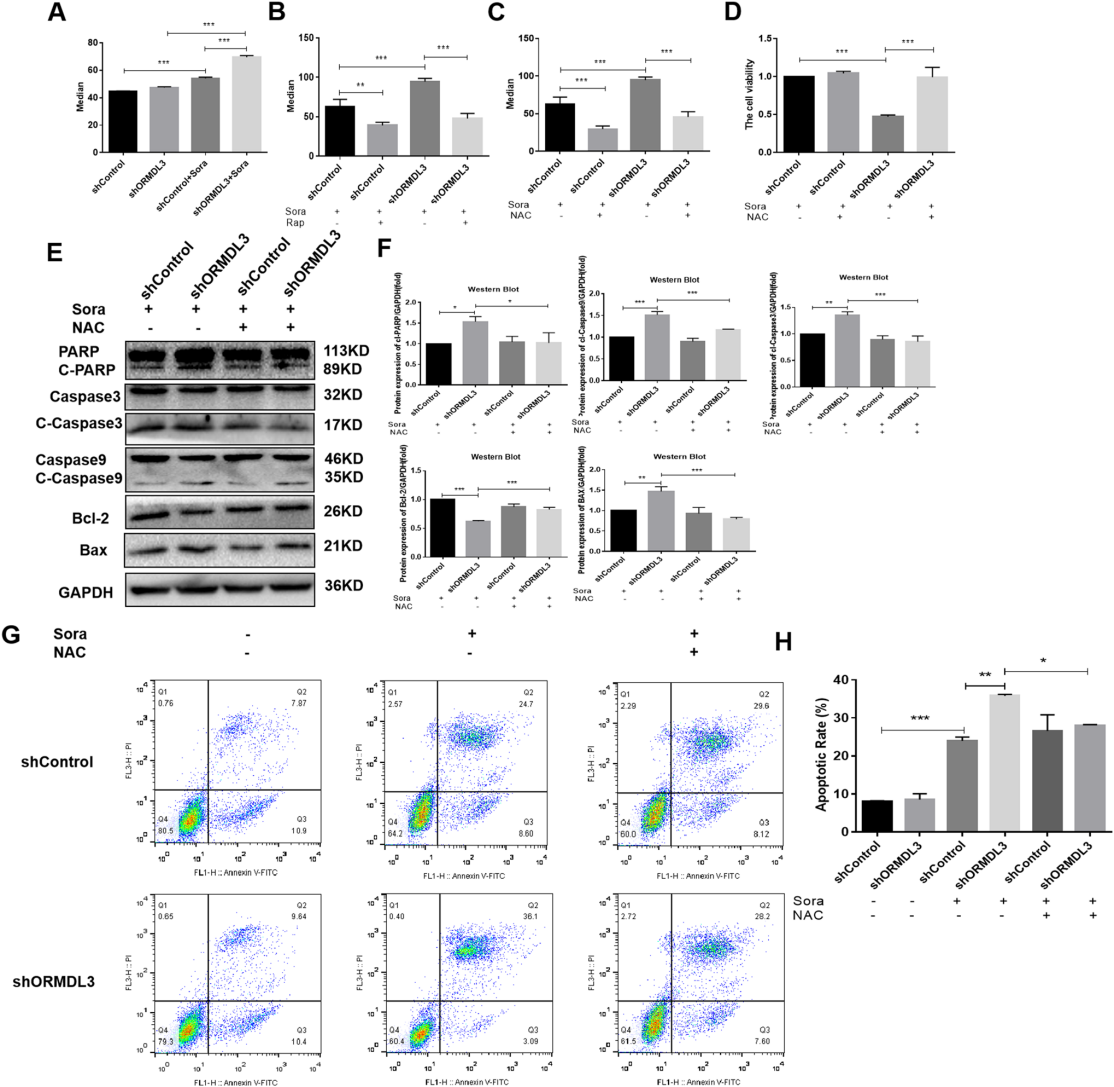
Increase in ROS levels by ORMDL3 silencing, which promotes ROS-mediated apoptosis. **A** Flow cytometry was used to detect the ROS level of ORMDL3-silenced HCC cells.** B** Flow cytometry was used to detect the ROS levels of HCC cells in each group treated with Rap (100 nM) as an autophagy activator, and **C** the ROS levels of HCC cells SMMC-7721 treated with NAC (10 mM). **D** Changes of HCC cell viability after administration of oxidative stress inhibitor NAC (10 mM). **E**, **F** Western Blot assay was used to detect the expression levels of Caspase 3, Caspase 9, PARP, Bax, and Bcl-2 proteins in ORMDL3-silenced human HCC cells SMMC-7721 after the use of oxidative stress inhibitor NAC and gray value analysis. **G** Annexin V-FITC/PI apoptosis kit was used for flow cytometry analysis to determine the apoptosis levels of ORMDL3-silenced groups. **H** Histogram of flow analysis data. Statistical analysis was performed using one-way ANOVA. *P < 0.05, **P < 0.01, ***P < 0.001. The data are presented as the means ± SD (*n* = 3)

In the above studies, it was found that silencing ORMDL3 can inhibit the autophagy of liver cancer cells induced by sorafenib, and it is known that autophagy is closely related to oxidative stress, so, changes in the oxidative stress level of cells were detected after silencing ORMDL3. The results showed that under the action of sorafenib, the level of ROS in silenced ORMDL3 liver cancer cells was significantly higher than that of the control group (Fig. 4A), that is, silencing ORMDL3 can make the level of oxidative stress to increase in liver cancer cells. In combination with the autophagy activator, Rap, it was found that the ROS level in silenced ORMDL3 liver cancer cells was reduced (Fig. 4B), indicating that silencing ORMDL3 made the level of ROS in liver cancer cells to increase by inhibiting autophagy. For further research, MitoSOX Red Mitochondrial Superoxide Indicator was used to detect the mitochondrial superoxide level of ORMDL3-silenced hepatoma cells, and showed that mitochondrial superoxide levels were elevated in the presence of sorafenib compared with controls, and silencing of ORMDL3 resulted in higher levels of mitochondrial superoxide in hepatoma cells (Fig. S1 C, D). We used ATP kit to detect changes in mitochondrial energy of liver cancer cells, and the results showed that compared with the control group, the ATP level of cells was reduced under sorafenib treatment, and the ATP level of liver cancer cells with silenced ORMDL3 was lower (Fig. S1 E).

Next, it was tested whether the increase in ROS levels caused by ORMDL3 silencing affected the apoptosis of liver cancer cells. The effect of ROS inhibitor N-Acetyl-l-cysteine (NAC) on reducing ROS levels (Fig. 4C) was first tested, and then, the apoptosis of liver cancer cells was tested through the MTT experiment, Western Blot, and Annex V-FITC / PI apoptosis kit. The results of MTT showed that liver cancer cells with silenced ORMDL3 had a significantly reduced viability when compared with the control group, and the cell viability increased after combining with the oxidative stress inhibitor NAC (Fig. 4D). The results of Western Blot showed that the expression of Cleaved Caspase 9, Cleaved Caspase 3, and Cleaved PARP in ORMDL3-silenced HCC cells was increased, the expression of apoptotic protein Bax was increased, and the expression of anti-apoptotic protein Bcl-2 was decreased (Fig. 4E, F). When combined with the oxidative stress inhibitor, NAC, the expression levels of these apoptotic proteins were reversed. Apoptosis levels were measured by flow cytometry with the Annex V-FITC/PI Apoptosis Kit, and the results were consistent with those described above. Apoptosis levels were reversed after the use of the oxidative stress inhibitor NAC (Fig. 4G, H). In summary, these experimental results indicate that silencing ORMDL3 can increase ROS-mediated apoptosis of liver cancer cells by inhibiting autophagy.

### Inhibition of autophagy through the PERK-ATF4-Beclin1 pathway by silencing ORMDL3

**Fig. 5:**
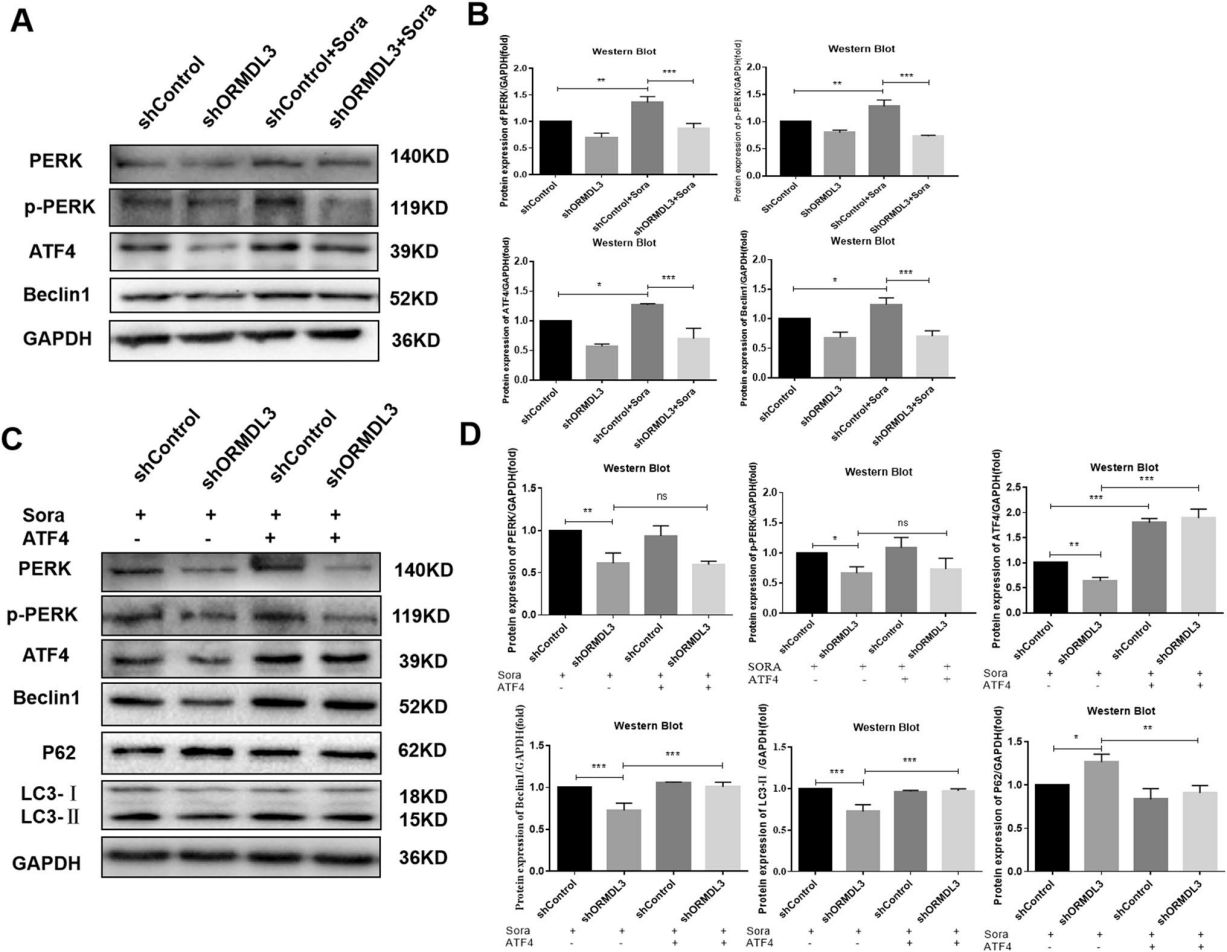
Inhibition of autophagy through the PERK-ATF4-Beclin1 pathway by silencing ORMDL3. **A**, **B** Western Blot assay was performed to detect the protein expression levels of PERK, p-PERK, ATF4, Beclin1 in ORMDL3-silenced human HCC cells and grayscale analysis. **C, D** Western Blot assay was performed to detect the protein expression levels of PERK, p-PERK, ATF4, Beclin1, P62, and LC3 after overexpression of ATF4 in ORMDL3-silenced human HCC cells SMMC-7721 and grayscale analysis. Statistical analysis was performed using one-way ANOVA. *P < 0.05, **P < 0.01, ***P < 0.001. The data are presented as the means ± SD (*n* = 3)

Inhibition of the PERK-ATF4 pathway may further reduce the expression of Beclin1, an important protein involved in autophagy, thus, increasing the sensitivity of sorafenib to HCC cells by inhibiting autophagy [26]. Therefore, it was investigated whether silencing ORMDL3 can also affect the PERK-ATF4 pathway to inhibit autophagy and increase sorafenib sensitivity to HCC cells. Western Blot analysis of the expression of PERK, p-PERK, ATF4, Beclin1 showed that when compared with the control group, the expression of PERK, p-PERK, ATF4, and Beclin1 was significantly increased after sorafenib treatment (Fig. 5A, B), which was due to the induction of ERS and autophagy by sorafenib. The expression of PERK, p-PERK, ATF4, and Beclin1 decreased in ORMDL3-silenced HCC cells after sorafenib treatment, suggesting that ORMDL3 silencing can inhibit the expression of PERK, p-PERK, ATF4, and Beclin1 proteins (Fig. 5A, B).

Next, it was further investigated whether ORMDL3 silencing inhibits PERK-ATF4 protein expression and then affected autophagy. The results showed that the deletion of ORMDL3 inhibited the expression of PERK, p-PERK, ATF4, Beclin1, and autophagy when compared with the control group. However, when ATF4 was overexpressed, PERK, p-PERK protein levels did not change significantly in ORMDL3 silencing group, while Beclin1 and LC3 protein levels increased and P62 protein levels decreased, indicating that ATF4 overexpression reversed the decrease in autophagy caused by silencing ORMDL3 (Fig. 5C, D). In conclusion, silencing ORMDL3 inhibited the expression of PERK-ATF4 and Beclin1, an important autophagy protein, thereby inhibiting the level of autophagy.

### In vivo study—establishment of subcutaneous tumor transplantation model in mice

**Fig. 6:**
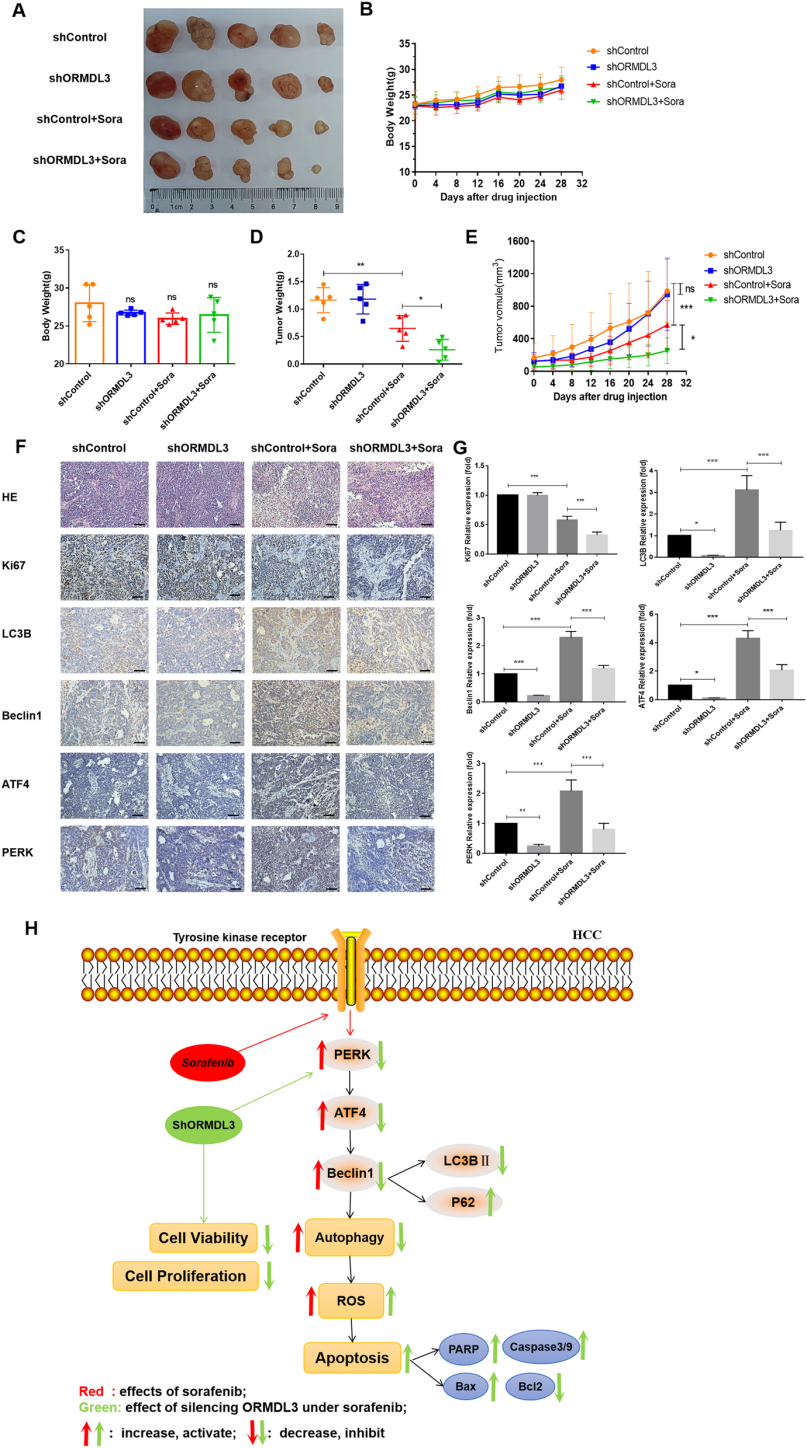
In vivo study—establishment of subcutaneous tumor transplantation model in mice. **A** Nude mice were divided into groups, and subcutaneously transplanted tumor models were constructed. The mice were given drugs every 2 days, and changes in body weight and tumor volume were recorded. On the 28th day, the mice were sacrificed and subcutaneous tumor masses were removed for analysis.** B** Mice were weighed every two days to make 28-day weight changes after drug injection. **C** The weight data of mice before death were obtained. **D** Tumor weight was detected after the mice were sacrificed. **E** Tumor growth trend analysis. Statistical analysis was performed using one-way ANOVA. ^ns^*P* > 0.05, *P < 0.05, **P < 0.01, ***P < 0.001. The data are presented as the means ± SD (*n* = 5). **F** H&E staining was performed to observe the tumor tissue sections. Immunohistochemical evaluation of Ki67, LC3B, Beclin1, ATF4, and PERK expressions in xenograft tumors of nude mice with different treatments. The brown expression was positive. Scale bar, 200 μM. **G** Image ProPlus (IPP) was used to analyze the expressions of Ki67, LC3B, Beclin1, ATF4, and PERK in immunohistochemical images. Statistical analysis was performed using one-way ANOVA. *P < 0.05, **P < 0.01,***P < 0.001. The data are presented as the means ± SD (*n* = 5). **H** Schematic diagram of the mechanism by which silencing ORMDL3 increases the sensitivity of HCC cells to sorafenib. Sorafenib acts on tyrosine kinase receptors of HCC cells, induces endoplasmic reticulum stress, and leads to increased levels of autophagy and ROS. Under the action of sorafenib, silencing of ORMDL3 can inhibit the PERK-ATF4-Beclin1 pathway, thereby inhibiting autophagy, increasing ROS levels, and increasing ROS-mediated apoptosis of liver cancer cells

We constructed a subcutaneous xenotransplantation model in nude mice, and the subcutaneous tumor masses were removed after the mice were sacrificed (Fig. 6A). According to the statistics of the bodyweight trends of mice (Fig. 6B) and the mice body weight on the 28th day (Fig. 6C), no significant difference was found between each group, indicating that the mice were in good and stable condition. Tumor weights were measured in each group and the results showed that there is no significant difference in tumor weight between the control group and the ORMDL3-silenced group. The tumor weight was significantly reduced by using sorafenib. Moreover, the tumor weight was significantly lower in the group with sorafenib after silencing ORMDL3, as compared to the group with sorafenib alone (Fig. 6D). The volume of mouse tumors was measured every two days and a data graph was made (Fig. 6E). It can be seen that the tumor volume of the mice increased gradually over time, and the group treated with no sorafenib increased far more than the group treated with sorafenib. The tumor volume growth rate of the control group and the ORMDL3-silenced group was the fastest, followed by the sorafenib group, and finally, the sorafenib group with ORMDL3 silenced. In summary, it is shown that sorafenib can inhibit the growth of hepatoma in mice, and silencing the expression of ORMDL3 in liver cancer cells can increase the inhibitory effect of sorafenib on the growth of hepatoma in mice.

Next, hematoxylin–eosin staining was used to observe the subcutaneous tumor tissue, and immunohistochemical staining was used to detect the ki67 proliferation index. When compared with the control group, there was no significant difference for the ki67 positive rate in ORMDL3-silenced group, while the ki67 positive rate in the two groups given sorafenib was significantly reduced. The positive rate of ki67 in the sorafenib group with ORMDL3 deletion was lower than that in the sorafenib group, indicating that sorafenib inhibited the proliferation of mouse hepatocellular tumor, which was enhanced by silencing ORMDL3. Positive rates of LC3B, Beclin1, ATF4, and PERK were also detected in subcutaneous tumor tissues, and the results are consistent with the above. Silencing ORMDL3 reduced the expressions of LC3B, Beclin1, ATF4, and PERK, inhibited the PERK-ATF4-Beclin1 pathway, and inhibited autophagy (Fig. 6F, G). In conclusion, subcutaneous tumor transplantation experiment showed that sorafenib could inhibit the growth of mouse hepatocellular tumors, while silencing HCC cell ORMDL3 could increase the inhibitory effect of sorafenib on the growth of mouse hepatocellular tumors.

## Discussion

Many studies have shown that ORMDL3 is associated with genetic susceptibility and the potential pathogenesis of asthma [27]. In addition to being highly correlated with asthma, ORMDL3 is also involved in many important signal transduction processes such as regulating cell growth, differentiation, senescence, and programmed cell death, thus playing an important role in many diseases [18, 19]. Although previous studies proved the relationship between ORMDL3 and ceramide in HepG2 hepatocarcinoma cells [21], its association with liver cancer is not completely clear. Therefore, based on the extensive influence of the ORMDL3 gene, it was explored whether the regulation of the ORMDL3 gene can affect the sensitivity of HCC cells to sorafenib. The current study showed that silencing ORMDL3 increased the inhibitory effect of sorafenib on HCC cell viability and proliferation. Moreover, deletion of ORMDL3 can increase the apoptosis of HCC cells induced by sorafenib (Fig. 6H). Overall, this experimental study demonstrated that the ORMDL3 gene can affect the sensitivity of HCC cells to sorafenib. Although the role of ORMDL3 in sorafenib resistance of liver cancer has been found, there are few studies on the role of the ORMDL3 gene in the occurrence and development of liver cancer and its molecular mechanism, which requires more research and discussion. The polymorphism in 17q21 containing the ORMDL3 gene is associated with several well-known inflammatory diseases, and subsequent gene function studies have found that it is closely related to ERS, ceramide metabolism, inflammatory response, and autophagy [28].

Many studies have shown that autophagy is very important in mediating sorafenib resistance, and inhibition of sorafenib-induced autophagy can significantly enhance the cytotoxicity of sorafenib in HCC cells [29, 30]. These studies suggest that targeting autophagy-related pathways may be a good method to reverse sorafenib resistance in the clinical treatment of HCC. Meanwhile, ORMDL3 is also closely related to autophagy. For example, studies have shown that ORMDL3 may mediate autophagy through ATF6-Beclin1 pathway, and promote the survival of spleen B cells by promoting autophagy and inhibiting apoptosis [31]. In this study, by detecting the expression of autophagy-related proteins, it was also found that silencing ORMDL3 could inhibit sorafenib-induced autophagy. The ability of ORMDL3 to regulate autophagy may be an important reason for the change of sensitivity of HCC cells to sorafenib. Therefore, our further study also found that silencing ORMDL3 can increase the apoptosis of sorafenib-induced HCC cells by inhibiting autophagy (Fig. 6H). Autophagy and nuclear factor erythroid-2 related factor 2 (NRF2) signal suppression can increase production of ROS and ERS in pancreatic cancer cells [32]. Inhibition of autophagy promotes apoptosis of adipose tissue-derived stem cells (ADSCs) mediated by high glucose reactive oxygen species [33]. These studies have shown the autophagy and oxidative stress level of intimate relationship. Therefore, we explored the relationship between the inhibition of autophagy and oxidative stress level after silencing ORMDL3, and found that silencing ORMDL3 could increase ROS-mediated apoptosis of HCC cells by inhibiting autophagy. To further explore why ORMDL3 silencing increased ROS-mediated apoptosis of HCC cells in the presence of sorafenib, we detected mitochondrial superoxide levels and mitochondrial energy changes. In summary, in the presence of sorafenib, silencing ORMDL3 increased mitochondrial superoxide level and reduced mitochondrial energy of HCC cells, indicating that silencing ORMDL3 caused mitochondrial dysfunction in HCC cells, which may be an important reason for increasing the apoptosis of HCC cells induced by sorafenib, which also needs further verification and exploration.

It is well known that, in response to ERS, cells tend to activate an evolutionally conformed response called UPR, which has three important pathways, of which PERK is one [34]. PERK is oligomerized in the ER, induces autophosphorylation and activation of the kinase domain, subsequently, phosphorylating the eukaryotic translation initiation factor 2α (elF2α), and then selectively increasing the translation of ATF4 to induce gene expression to maintain ER homeostasis [35]. It has been found that inhibition of the PERK-ATF4 pathway can reduce the expression of Beclin1, an important protein involved in autophagy, thereby inhibiting autophagy increases the sensitivity of sorafenib to HCC cells [26, 36]. It was also found that ORMDL3 overexpression accelerated the phosphorylation of PERK mediated by toxic carotene [37]. In conclusion, the above findings indicate that the ORMDL3 gene, autophagy, and PERK-ATF4 in ERS are interlinked. Consistent with this, our study found that the expressions of PERK, ATF4, and Beclin1 decreased in ORMDL3-silenced HCC cells, regardless of whether sorafenib was used. When ATF4 is overexpressed, as compared to the ORMDL3-silenced group, PERK protein level did not change significantly, while Beclin1 and LC3 protein levels increased, and P62 protein decreased, indicating that overexpressed ATF4 reversed the decrease in autophagy caused by silencing ORMDL3. These results suggest that ORMDL3 can inhibit autophagy levels by inhibiting the PERK-ATF4 pathway and reducing the expression of Beclin1, an important protein formed by autophagy (Fig. 6H). It was further demonstrated that the PERK-eIF2α-ATF4 pathway is critical in detecting ERS and inducing autophagy to address misfolded protein accumulation through lysosomal degradation [38].

## Conclusion

In conclusion, the role of the ORMDL3 gene in sorafenib resistance of HCC was thoroughly investigated and described in the current study, showing that inhibition of the ORMDL3 gene increases the sensitivity of HCC cells to sorafenib. The study reveals a potential molecular mechanism that enhances the efficacy of sorafenib in HCC and may provide new approaches for better treatment design.

## Supplementary Information

Below is the link to the electronic supplementary material.

Supplementary file1 (TIF 492 kb)

## Data Availability

The original contributions presented in the study are included in the article/supplementary materials. Further inquiries can be directed to the corresponding author.

## Abbreviations

HCC: Hepatocellular carcinoma
ORMDL3: Serum orosomucoid1-like protein 3
ROS: Reactive oxygen species
PERK: Double-stranded RNA-dependent protein kinase-like endoplasmic reticulum kinase
ATF4: Activated transcription factor 4
ATF6: Activated transcription factor 6
NRF2: Nuclear factor erythroid-2 related factor 2
ER: Endoplasmic reticulum
UPR: Unfolded protein response
SPT: Serine palmitoyltransferase
ERS: Endoplasmic reticulum stress
qRT-PCR: Quantificational real-time polymerase chain reaction
CQ: Chloroquine diphosphate salt
Rap: Rapamycin
NAC: N-Acetyl-L-cysteine
eIF2α: Eukaryotic initiation factor 2
HE: Hematoxylin–eosin
ADSCs: Adipose tissue-derived stem cells
