# Supplementary figures and images for: Regulation of the sensitivity of hepatocarcinoma cells by ORMDL3, to sorafenib by autophagy
Source: Med Oncol. 2022 Aug 16;39(11):159. doi: 10.1007/s12032-022-01767-z (PMC9381447; doi:10.1007/s12032-022-01767-z)

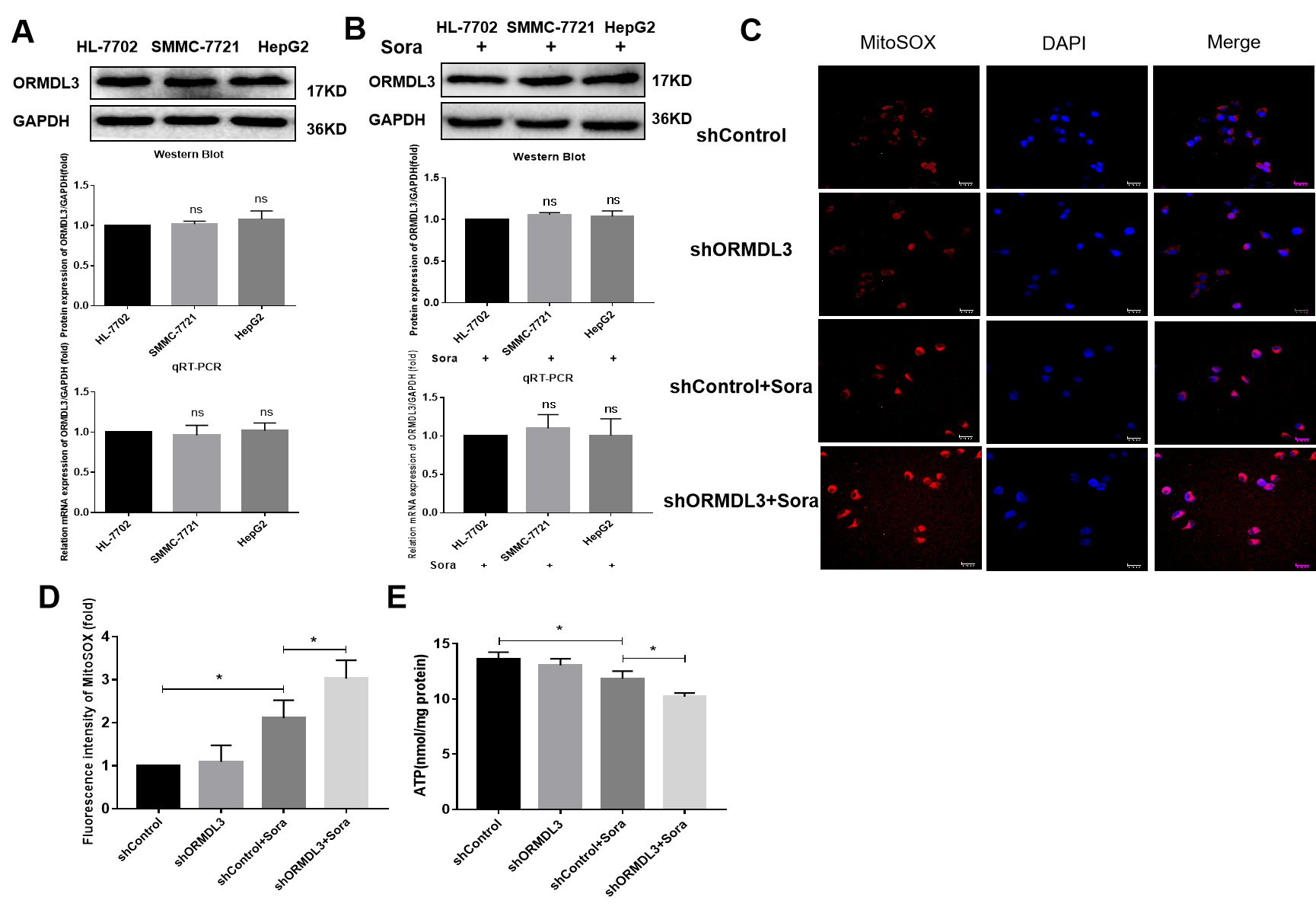

Supplement: Supplementary file 1 — Supplementary file1 (TIF 492 kb) [file 12032_2022_1767_MOESM1_ESM.tif]
